# Lsamp is implicated in the regulation of emotional and social behavior by use of alternative promoters in the brain

**DOI:** 10.1007/s00429-014-0732-x

**Published:** 2014-03-15

**Authors:** Mari-Anne Philips, Kersti Lilleväli, Indrek Heinla, Hendrik Luuk, Christian Ansgar Hundahl, Karina Kongi, Taavi Vanaveski, Triin Tekko, Jürgen Innos, Eero Vasar

**Affiliations:** 1 Department of Physiology, Institute of Biomedicine and Translational Medicine, University of Tartu, Tartu, 50411 Estonia; 2 Department of Developmental Biology, University of Tartu, Tartu, Estonia; 3 Department of Clinical Biochemistry, Bispebjerg Hospital, University of Copenhagen, Copenhagen, Denmark

**Keywords:** Lsamp, Alternative promoters, Limbic system, Sensory pathways, Anxiety, Social behavior

## Abstract

Limbic system-associated membrane protein (LSAMP) is a neural cell adhesion molecule involved in neurite formation and outgrowth. The purpose of the present study was to characterize the distribution of alternatively transcribed *Lsamp* isoforms in the mouse brain and its implications on the regulation of behavior. Limbic system-associated membrane protein 1b transcript was visualized by using a mouse strain expressing beta-galactosidase under the control of *Lsamp* 1b promoter. The distribution of *Lsamp* 1a transcript and summarized expression of the *Lsamp* transcripts was investigated by non-radioactive in situ RNA hybridization analysis. Cross-validation was performed by using radioactive in situ hybridization with oligonucleotide probes. Quantitative RT-PCR was used to study correlations between the expression of *Lsamp* isoforms and behavioral parameters. The expression pattern of two promoters differs remarkably from the developmental initiation at embryonic day 12.5. Limbic system-associated membrane protein 1a promoter is active in “classic” limbic structures where the hippocampus and amygdaloid area display the highest expression. Promoter 1b is mostly active in the thalamic sensory nuclei and cortical sensory areas, but also in areas that regulate stress and arousal. Higher levels of *Lsamp* 1a transcript had significant correlations with all of the measures indicating higher trait anxiety in the elevated plus-maze test. Limbic system-associated membrane protein transcript levels in the hippocampus and ventral striatum correlated with behavioral parameters in the social interaction test. The data are in line with decreased anxiety and alterations in social behavior in *Lsamp*-deficient mice. We propose that *Lsamp* is involved in emotional and social operating systems by complex regulation of two alternative promoters.

**Electronic supplementary material:**

The online version of this article (doi:10.1007/s00429-014-0732-x) contains supplementary material, which is available to authorized users.

## Introduction

Limbic system-associated membrane protein (LSAMP) is a neural cell adhesion molecule expressed on the neuronal dendrites and somata (Zacco et al. 1990) on structures known to be especially important for emotional and motivational functions (Heimer and Van Hoesen 2006; Levitt 1984). Recently, LSAMP has been linked with a spectrum of psychiatric disorders in humans. The levels of the LSAMP protein have been found to be approximately 20 % increased in the postmortem frontal cortex both in patients with schizophrenia and bipolar disorder (Behan et al. 2009). Polymorphisms in the *LSAMP* gene have been associated with depression (Koido et al. 2012) and *LSAMP* has been suggested to have a role in the neurobiology of male completed suicide (Must et al. 2008).

Functional studies have shown that LSAMP can promote or inhibit neurite outgrowth depending on interactions with other members of the IgLON family (Mann et al. 1998; Gil et al. 2002), indicating its prominent role in neurite formation and synaptogenesis (Hashimoto et al. 2009). Before second postnatal week of development, LSAMP is transiently also expressed in developing axons and growth cones (Horton and Levitt 1988) indicating importance in developing of the brain structures. However, the lack of obvious deviations in brain organization in both of the two independently created *Lsamp*-deficient mouse strains (Catania et al. 2008; Innos et al. 2011) suggests that LSAMP is mediating finely specialized aspects of circuit formation and maturation of the limbic system. Genetic deletion of the *Lsamp* gene in mice induced no detectable changes in sensory and motor development, but caused increased activity in novel environments and reduced anxiety-like behavior in both knockout models (Catania et al. 2008; Innos et al. 2011). Increased trait anxiety in rats has been shown to be related with increased level of the *Lsamp* transcript in the amygdaloid area, periaqueductal gray (Nelovkov et al. 2006), raphe, hippocampus and frontal cortex (Alttoa et al. 2010). Elevated levels of the *Lsamp* transcript in the amygdaloid area of rats have been associated with acute fear reaction (Koks et al. 2004) and fear conditioning (Lamprecht et al. 2009). The amino acid sequence of LSAMP is highly conserved among species. There is 99 % sequence identity between human and rodent LSAMP (Pimenta and Levitt 2004) and 91 % identity with chicken (Brümmendorf et al. 1997), indicating remarkable phylogenetic conservation of protein structure and associated functional properties. Growing evidence indicates that LSAMP is involved in the formation of anatomical substrate for emotional behavior both in rodents and humans.

Pimenta and Levitt (2004) reported revised genomic structure of the mouse *Lsamp* gene, demonstrating that, besides the well-known exon 1 (now referred as exon 1b), the *Lsamp* gene has an alternative exon 1 (currently exon 1a) located 1.6 Mbp upstream, and both of them have separate promoter sequences. The two-promoter structure is conserved and has been described in mouse, rat, human (Pimenta and Levitt 2004) and also in chicken (Brümmendorf et al. 1997). Limbic system-associated membrane protein distribution in the whole adult mammalian brain was originally described in rat using immunohistochemistry (Levitt 1984) and in situ hybridization (Reinoso et al. 1996). The anatomical distribution of the *LSAMP* transcript and protein has been extensively described in various species (Chesselet et al. 1991; Côté et al. 1995; Prensa et al. 2003; Yamamoto and Reiner 2005), but the anatomical distribution of the alternative transcripts has not been reported. The purpose of the present study was to characterize the distribution of the two alternatively transcribed *Lsamp* isoforms in the mouse brain and to provide an initial analysis of the developmental activation of *Lsamp* promoters. Additionally, we investigated the relation between *Lsamp* expression and the regulation of emotional and social behavior.

## Methods

### Non-radioactive in situ RNA hybridization analysis with digoxigenin-UTP

Mouse *Lsamp* cDNA fragments were cloned from a cDNA pool from C57BL/6 mouse hippocampus and inserted into *pGEM7*-Zf(+) vector (Promega) to create an in situ probe. cDNA fragment specific for 1a promoter (400 bp) consisted of 1a-specific 5′UTR, exon 1a and exon 1a′. Universal *Lsamp* probe (567 bp) consisted of a cDNA fragment consisting of exons 2–6. RNA in situ hybridization on free-floating PFA-fixed 40 μm mouse brain cryosections using digoxigenin-UTP (Roche) labeled *Lsamp* sense and antisense RNA probes was performed as described previously (Braissant and Wahli 1998). As a major modification, active DEPC treatment was avoided and 0.25 % Triton X-100 was added to the PBS to improve probe penetration.

### Radioactive in situ hybridization with oligonucleotides

Antisense 40′mer DNA oligonucleotide probes complementary to mouse *Lsamp* gene (Accession No. uc007zfr.1, UCSC Genome Browser, genome.ucsc.edu) Lsamp1a (5′-accccagcacccagacgctgtgcagccagtaggtcctcat-3′), Lsamp1b (5′-gaagaaggcagagcagtctcagtaggaccagcggcaactg-3′) and LsampUNI (5′-agagcatggcgcttctccagctcaacccgagggtccagag-3′) were labeled with ^33^P-dUTP by use of terminal transferase (Sigma-Aldrich, Europa). Free-floating in situ hybridization was carried out essentially as described previously (Hundahl et al. 2010).

### *Lsamp*-deficient mice with beta-galactosidase knock-in

Detailed description of the generation of the *Lsamp*-deficient mice with a LacZ transgene can be found in Innos et al. (2011). Briefly, exon 1b of mouse *Lsamp* gene was replaced by an in-frame NLSLacZNeo cassette resulting in insertion of gene encoding beta-galactosidase immediately after *Lsamp* 1b promoter. X-Gal staining for detecting the distribution of 1b promoter specifically produced beta-galactosidase and NeuN immunostaining was performed as described previously (Luuk et al. 2008). The brains were cut into 100 μm coronal sections and transferred to glass slides. The contours in Fig. 4c, f and abbreviations in all the figures representing anatomical data have been adopted from the mouse brain atlas (Franklin and Paxinos 1997). For embryonic brains, the embryos were dissected from timed matings as in Philips et al. (2009). E13.5 (embryonic day 13.5) and E15.5 brains and/or embryos were fixed in 4 % PFA/PBS at 4 °C overnight for in situ hybridization, or for 30 min for X-gal staining. For cutting 50-μm vibratome sections, the stained (E13.5 and E15.5) specimens were inserted into 1 ml of 0.5 % gelatine/30 % BSA/20 % sucrose/PBS, wherein 140 μl of 25 % glutaraldehyde was added immediately before insertion and incubated for 10 min. The sections were mounted into 70 % glycerol and microphotographed.

### Experimental animals for behavioral studies and quantitative real-time PCR analysis

Male C57BL/6 strain mice were used for all behavioral studies. In the first part of the behavioral studies we investigated the correlations between general behavioral profile and *Lsamp* expression that was measured 10 days after behavioral testing. All mice (*n* = 15) were subjected to a battery of three behavioral tests: elevated plus-maze test (day 0), locomotor activity test (day 5), and social interaction test (day 10), with less invasive tests preceding more stressful tests. Testing began at age 60 days (2 months). The motility box was carried out as described previously (Innos et al. 2013) and four behavioral parameters were recorded: (1) time spent moving in seconds (move, s); (2) distance travelled in meters (distance, m); (3) time spent in the center (time center, s); and (4) time spent in the corners (time corner, s). The plus-maze experiment was performed as described in Philips et al. (2010) and eight behavioral parameters were recorded: (1) the number of closed arm entries; (2) the number of open arm entries; (3) the ratio between open and closed arm entries; (4) the latency to enter open arm (latency, s); (5) time spent on open arms; (6) the number of protected head-dips; (7) the number of unprotected head-dips; and (8) the number of stretch-attend postures (SAPs). The social interaction test was carried out as described previously (Innos et al. 2012), briefly: 14 mice were matched into 7 pairs of two unfamiliar mice according to the bodyweight and (1) the time spent sniffing the partner’s anogenital area (anogenital sniffing, s) and (2) the time spent sniffing other body regions (sniffing other body parts, s) were recorded separately. These two measures were also summarized for (3) the time of total social sniffing for each animal (time of social sniffing, s). The animals were decapitated 10 days after the last experiment and three parts were dissected from the brains: the ventral striatum (including nucleus accumbens and olfactory tuberculi), hippocampus and temporal lobe (including temporal cortex and lateral, central and medial nuclei of amygdala).

In the second part of the behavioral studies, we investigated the influence of acute fear reaction on the expressional activity of the *Lsamp* gene. In order to induce acute fear reaction, fear conditioning was carried out by means of a computer-controlled Multi Conditioning System (TSE). Sixteen mice were divided into three groups: “Naïve” (*n* = 5); “Pre-conditioning” (*n* = 5) and “Conditioned fear” (*n* = 6). Training for the “Conditioned fear” and “Pre-conditioning” groups was performed in a dimly illuminated (15 lx) acrylic cage (30 × 30 × 30 cm) with stainless steel rod floor. Between subjects the cages were cleaned with isopropanol. On the first day after 150-s acclimation period animals received six trials with the following stimuli: 15 s tone (12 kHz; 70 dB) and bright light (pulsing at 200 ms) were terminated by a 2-s electric shock (0.6 mA) during which the light was constant. Inter-trial interval was 120 s (±50 %). After the last trial the animals were returned to their home cages. On the second day, animals in the “Conditioned fear” group were placed into the conditioning cages and exposed to similar stimuli without an electric shock for about 45 min (20 trials). The animals were killed immediately afterwards. We chose a 45-min duration as it has been shown that neuronal stimulation induces gene expression between timepoints 30–60 min (Lamprecht et al. 2009). “Naïve” and “Pre-conditioning” groups received no treatment on the second day. The hippocampus and temporal lobe (including the temporal cortex and the lateral, basolateral, central and medial nuclei of the amygdala) were dissected from the brains.

All the experiments were performed in accordance with the EU guidelines directive 86/609/EEC) and permit (No. 59, September 5, 2006) from the Estonian National Board of Animal Experiments.

### qRT-PCR analysis of *Lsamp* expression in mouse brain areas

The tissue samples were frozen in liquid nitrogen. Dissection of the mouse brain was performed according to coordinates obtained from the mouse brain atlas (Franklin and Paxinos 1997). Limbic system-associated membrane protein mRNA level was determined by quantitative real-time PCR (qRT-PCR). Total RNA was extracted individually from each brain structure by using Trizol^®^ reagent (Invitrogen, USA) according to the manufacturer’s protocol. First strand cDNA was synthesized by using Random Hexamers (Applied Biosystems) and SuperScript™ III Reverse Transcriptase (Invitrogen, USA). TaqMan Assay was designed for the detection of 1a- and 1b-specific transcripts. FAM-MGB-probe AACCGAGGCACGGACAAC was used with universal reverse primer combined with alternative forward oligos specific for either 1a isoform or 1b isoform. For mRNA quantification of the immediate-early gene c-fos, a Taqman assay from Applied Biosystems was employed (Mm00487425_m1). The detailed sequences of primers and probe for housekeeping gene hypoxanthine guanine phosphoribosyl transferase (*Hprt*-*1*) have been described in Areda et al. (2005). TaqMan^®^ Universal PCR Master Mix was used in the ABI Prism 7900HT Sequence Detection System (Applied Biosystems, USA). Reactions were carried out in 10-μl reaction volumes in four replicates.

### Data analysis

The analysis of qRT-PCR data was performed as described earlier (Raud et al. 2009). Briefly, qRT-PCR data in Fig. 3 is presented in linear scale, calculated as 2^−ΔCT^, where ΔCT is the difference in cycle threshold (CT) between the target gene (*Lsamp*) and housekeeper gene *Hprt*-*1* (VIC-MGB). All data were analyzed using Statistica version 8.0 (StatSoft, Inc., USA). As the behavioral scores were not normally distributed, Spearman’s rank-order method was used for the calculation of correlation coefficients. In the fear conditioning study, one-way ANOVA (conditioning type as grouping variable) was performed. Tukey HSD post hoc analysis was used when applicable after statistically significant ANOVA. Data are presented as mean ± SEM.

## Results

### *Lsamp* 1a promoter activity predominates in “classic” limbic structures

**Fig. 1:**
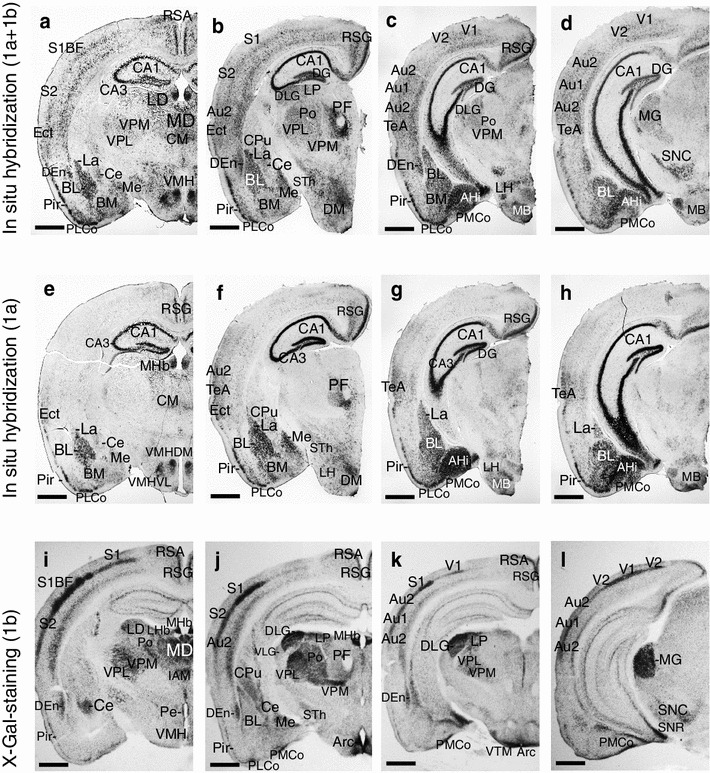
Non-radioactive in situ RNA hybridization analysis with digoxigenin-UTP representing universal *Lsamp* transcript (**a**–**d**) or *Lsamp* 1a transcript (**e**–**h**) and X-Gal staining expressing 1b promoter activity (**i**–**l**). *AHi* amygdalohippocampal area, *Arc* arcuate hypothalamic nucleus, *Au1/Au2* primary/secondary auditory cortex, *BL/BM* basolateral/basomedial amygdaloid nucleus, *CA1/CA3* CA1/CA3 field of hippocampus, *Ce* central nucleus of amygdala, *CM* central medial thalamic nucleus, *CPu* caudate putamen, *DEn* dorsal endopiriform nucleus, *DG* dentate gyrus, *DLG/VLG* dorsal/ventral lateral geniculate nucleus, *DM* dorsomedial hypothalamic nucleus, *Ect* ectorhinal cortex, *Ins* insular cortex, *La* lateral amygdaloid nucleus, *LHb/MHb* lateral/medial habenular nucleus, *LD/MD* laterodorsal/mediodorsal thalamic nucleus, *LP* lateral posterior thalamic nucleus, *LH* lateral hypothalamic area, *MB* mammillary bodies, *Me* medial amygdaloid nucleus, *MG* medial geniculate nucleus, *Pe* periventricular hypothalamic nucleus, *Pir* piriform cortex, *PF* parafascicular thalamic nucleus, *PLCo/PMCo* posterolateral/posteromedial cortical amygdaloid nucleus, *Po* posterior thalamic nuclear group, *RSA/RSG* retrosplenial agranular/granular cortex, *S1/S2* primary/secondary somatosensory cortex, *S1BF* S1, barrel field, *SNC/SNR* substantia nigra, compact/reticular part, *STh* subthalamic nucleus, *TeA* temporal association cortex, *V1/V2* primary/secondary visual cortex, *VMH,* *VMHDM/VMHVL* ventromedial thalamic nucleus, dorsomedial part/ventrolateral part, *VPM/VPL* ventral posteromedial/posterolateral thalamic nucleus, *VTM* ventral tuberomammillary nucleus. *Scale bar* 1 mm

**Fig. 2:**
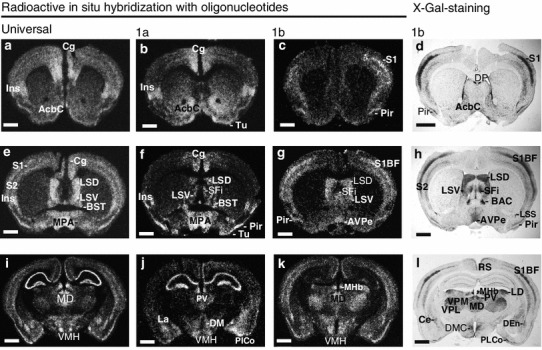
Radioactive in situ hybridization with oligonucleotides. Universal probe (**a**, **e**, **i**); 1a transcript-specific probe (**b**, **f**, **j**) or 1b specific probe (**c**, **g**, **k**) has been used and complementary X-Gal staining in respective brain areas representing 1b-specific staining (**d**, **h**, **l**) has been shown. *AcbC* accumbens nucleus, core, *AVPe* anteroventral periventricular hypothalamic nucleus, *BAC* bed nucleus of the anterior commissure, *BST* bed nucleus of stria terminalis, *Ce* central nucleus of amygdala, *Cg* cingulate cortex, *DP* dorsal peduncular cortex, *DM* dorsomedial hypothalamic nucleus, *Ins* insular cortex, *LSD/LSV* lateral septal nucleus, dorsal part/ventral part, *LSS* lateral stripe of striatum, *LD/MD* laterodorsal/mediodorsal thalamic nucleus, *MPA* medial preoptic area, *Pir* piriform cortex, *PV* paraventricular thalamic nucleus, *S1/S2* primary/secondary somatosensory cortex, *S1BF* S1, barrel field, *SFi* septofimbrial nucleus, *Tu* olfactory tubercle, *VPM/VPL* ventral posteromedial/posterolateral thalamic nucleus. *Scale bar* 1 mm

Limbic system-associated membrane protein 1a transcript is intensively and specifically expressed in the brain areas that are commonly considered to be limbic structures (Heimer and Van Hoesen 2006; Morgane et al. 2005). Transcript 1a-specific staining is pronounced in the cingulate cortex (Cg, Fig. 2b, f; supplementary Fig. S1e), insular cortex (Ins, Fig. 2b, f; Fig. S1e, f), prelimbic cortex (PrL, Fig. S1e) and infralimbic cortex (IL, Fig. S1e). Extensive 1a-specific staining can be seen in the hippocampal formation (CA1, CA3 and DG; Figs. 1e–h, 2j), amygdalohippocampal area (AHi, Fig. 1g, h), lateral amygdaloid nucleus (La, Figs. 1e–g, 2j, 4c), basolateral (BL, Fig. 1e–h and BLA, Fig. 4c) and basomedial (BM, Fig. 1e, f) amygdaloid nuclei, medial amygdaloid nucleus (Me, Fig. 1f) and posterolateral (PLCo, Fig. 1e–g) and posteromedial (PMCo, Fig. 1g, h) cortical amygdaloid nuclei. Transcript 1b-specific X-Gal staining and in situ signal are much weaker in these areas. However, there is moderate 1b-specific staining in the central amygdaloid nucleus (Ce, Figs. 1i, 2l, 4f) and cortical amygdaloid nuclei (PLCo/PMCo, Fig. 1j–l). Expression of 1b isoform in the hippocampal formation is moderate and homogeneous (Figs. 1i–l, 2k, l; Fig. S1m). qRT-PCR results confirm the prevalent expression of 1a transcript in the hippocampal area and temporal lobe (Fig. 4e).

### *Lsamp* 1b promoter activity is prevalent in the sensory nuclei and primary cortex areas

Many of the sensory systems are distinguished by 1b promoter-specific staining. In the major afferent pathways for somatosensory information, intense *Lsamp* 1b-specific staining is seen in the ventral posterior lateral thalamic nucleus (VPL; Figs. 1i–k, 2k, l) and primary somatosensory cortex (S1, Figs. 1j, k, 2c, d, g, k). The expression signal is the highest in the barrel field (S1b, f, Figs. 1i, 2h, l). There is moderate 1b transcript-specific staining in the gracile, cuneate (Cu, supplementary Fig. S1s) and spinal trigeminal (Sp5, Fig. S1s) nuclei and strong staining in the laterodorsal (Bezdudnaya and Keller 2008) thalamic nucleus (LD, Fig. 1i). In the ascending auditory pathway there is strong 1b-specific staining in the dorsal and ventral cochlear nuclei (DC and VC, respectively, Fig. S1o, p) and moderate 1b-specific staining in the superior olivary complex (SOC, Fig. S1p) and trapezoid body (Tz, Fig. S1p). Isoform 1b staining is strong in the nuclei of lateral lemniscus (LL, Fig. S1n), in the inferior colliculus (IC, Fig. S1o), in the medial geniculate nucleus (MG, Fig. 1l) and also in the primary and secondary (Au1/Au2, Fig. 1k, l) auditory cortex. In the visual pathway there is intensive 1b transcript-specific staining in the dorsal lateral geniculate thalamic nucleus (DLG, Fig. 1j, k; Fig. S1m) and primary visual cortex (V1, Fig. 1l; Fig. S1n). Strong 1b-specific X-Gal staining is also found in other brain areas receiving major projections from the retina: the superior colliculus (SC, Fig. S1n) and suprachiasmatic nucleus (SCh, Fig. S1i, j, l), and weak in the ventral lateral geniculate thalamic nucleus (VLG, Fig. 1j; Fig. S1m). In the sensory areas of the cortex, *Lsamp* 1b staining forms two distinct lines corresponding to layers 4 and 6 of the cortex (Lein et al. 2007) as estimated by comparing X-Gal staining (Fig. S1d) with NeuN immunoreactivity in the cortex (Fig. S1c). Staining reflecting promoter 1a activity is weak in the sensory areas of the cortex (Fig. S1a), and summarized expression of both isoforms reveals two moderate but distinct lines (Fig. S1b).

Both 1a and 1b promoters are expressed in brain areas involved in the processing of gustatory and olfactory information. In the gustatory system, 1b-specific staining is strong in the ventral posteromedial nucleus (VPM, Fig. 1i–k) and weak in the solitary nucleus (Sol, supplementary Fig. S1s). In the insular cortex only 1a isoform is expressed (Ins, Fig. 2b, f; Fig. S1e, f). In the olfactory system, the activity of 1b promoter is remarkable in the mediodorsal (Tham et al. 2009) thalamic nucleus (MD, Figs. 1i, 2k, l), and prevalent in the olfactory bulb (data not shown) and entorhinal cortex (Ent, Fig. S1n). The expression of 1a promoter is distinct in the nucleus of the lateral olfactory tract (LOT, Fig. S1k), and it dominates over 1b signal in the olfactory tubercle (Tu, Fig. 2b) and piriform cortex (Pir, Fig. 1e–h).

### Differences in the activity of *Lsamp* 1a and 1b promoters in adult brain

Thalamic and hypothalamic nuclei are distinguished by the isoform-specific expression of *Lsamp*. Only two hypothalamic nuclei display activity for both 1a and 1b promoters: the paraventricular nucleus (Pa, supplementary Fig. S1k, l) and mammillary bodies (MB, Fig. 1c, g, k). High expression of 1a isoform is seen in the ventromedial hypothalamic nucleus, namely in the ventrolateral (VMHVL, Fig. 1e) and dorsomedial parts (VMHDM, Fig. 1e); weak 1b-specific staining is present in the anterior part of the VMH (Fig. 2k). Promoter 1a is active in the dorsomedial hypothalamic nucleus (DM, Fig. 1f), while the activity of 1b promoter is limited to the compact part of the DM (DMC, Fig. 2l). Strong 1a promoter-specific expression can be seen in the medial preoptic area (MPA, Fig. 2b), including medial preoptic nucleus, and also in the ventromedial preoptic nucleus (VMPO, Fig. S1f). Promoter 1a-specific staining is moderate in the anterolateral (LA, Fig. S1k) and lateral hypothalamus (LH, Fig. 1f, g; Fig. S1k, l). Strong 1b promoter-specific expression can be detected in the periventricular hypothalamic nucleus (Pe, Fig. 1i; Fig. S1l), anteroventral periventricular nucleus (AVPe, Fig. 2h), suprachiasmatic nucleus (SCh, Fig. S1i, j, l), supraoptic nucleus (SO, Fig. S1l) and arcuate nucleus (Arc, Fig. 1j, k).

Promoter 1a is specifically active in the anterior thalamus: the anteroventral thalamic nucleus (AV, supplementary Fig. S1g), reticular thalamic nucleus (Rt, Fig. S1k) and central medial thalamic nucleus (CM, Fig. 1e). Weak 1a-specific expression can be detected in the anteromedial thalamic nucleus (AM, Fig. S1g). Isoform 1b-specific staining can be seen in the sensory thalamic nuclei as described above, but also in the posterior thalamic nuclei (Po, Fig. 1i, j), lateral habenular nucleus (LHb, Fig. 1i), lateral posterior thalamic nucleus (LP, Fig. 1j, k), paratenial thalamic nucleus (PT, Fig. S1h) and reuniens thalamic nucleus (Re, Fig. S1l). There are numerous thalamic nuclei where both 1a and 1b promoters are active: the anterodorsal thalamic nucleus (AD, Fig. S1g, h), paraventricular thalamic nucleus (PV, Fig. S1k, l), paracentral thalamic nucleus (PC, Fig. S1g, h), parafascicular thalamic nucleus (PF, Fig. 1b, f, j), medial habenular nucleus (Mhb, Fig. 1a, e, i) and subthalamic nucleus (STh, Fig. 1b, f, j).

Alternative expression of *Lsamp* 1a and 1b promoters can be seen throughout the brain. Both 1a and 1b promoters are active in the dorsal (LSD, Fig. 2e–h) and ventral (LSV, Fig. 2e–h) part of the lateral septal nucleus, septofimbrial nucleus (SFi, Fig. 2f–h), subfornical organ (SFO, Fig. S1g, h) and retrosplenial granular cortex (RSG, Fig. 1e–g, i–k), whereas only 1b promoter is active in the retrosplenial agranular cortex (RSA, Fig. 1i–k). On the level of anterior commissure, 1b promoter is active in the bed nucleus of anterior commissure (BAC, Fig. 2h); 1a transcript is prevalent in the bed nuclei of stria terminalis (BST, Fig. 2f, Fig. S1f) and in the core of the nucleus accumbens (AcbC, Fig. 2b). Isoform 1b-specific staining is present in the dorsal peduncular cortex (DP, Fig. 2d), caudate putamen (CPu, Fig. 1j), dorsal endopiriform nucleus (Den, Fig. 1i–k), claustrum (Cl, Fig. 2h) and lateral stripe of striatum (LSS, Fig. 2h). The expression of the *Lsamp* transcript in the cerebellum is mostly initiated from promoter 1b, which is abundant in the Purkinje cell layer (Pc, Fig. S1r). There is moderate 1b expression in the molecular layer (Mc, Fig. S1r) and weak 1a expression in the granule cells (Gc, Fig. S1q). Limbic system-associated membrane protein expression is moderate in the spinal cord: both 1a (Fig. S1t) and 1b (Fig. S1u) isoforms are expressed in the ventral and dorsal horns. A detailed overview of the estimated intensities of *Lsamp* 1a, 1b and summarized transcripts in different brain areas has been presented in supplementary Table S2.

### Differences in the activity of *Lsamp* 1a and 1b promoters in embryonic brain

**Fig. 3:**
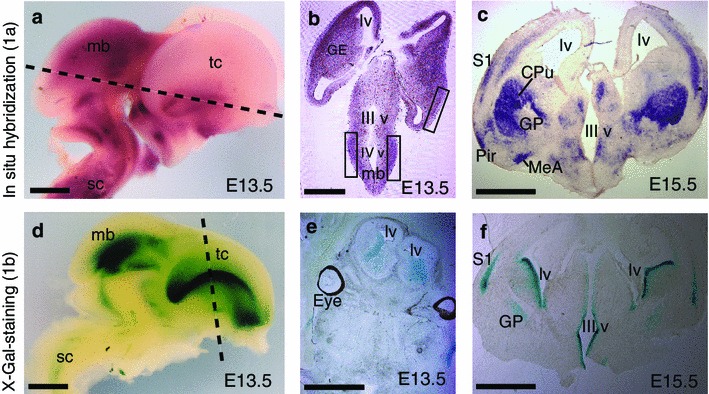
Distribution of *Lsamp* 1a and 1b transcripts during development. Non-radioactive in situ RNA hybridization analysis representing *Lsamp* 1a transcript (**a**–**c**) and X-Gal staining expressing 1b promoter activity (**d**–**f**) in the embryonic mouse brain. *CPu* caudate putamen, *GE* ganglionic eminence, *GP* globus pallidus, *lv* lateral ventricle, *mb* midbrain, *Pir* piriform cortex, *S1* primary somatosensory cortex, *sc* spinal cord, *tc* telencephalon, *III v* third ventricle, *IV v* fourth ventricle. *Dashed lines* in **a** and **d** represent approximate cross-sections for **b** and **e**, respectively. *Scale bar* 1 mm

We detected the first signals for both promoters of the *Lsamp* gene at around E12.5. Limbic system-associated membrane protein 1a transcript is firstly activated in the midbrain, being prominent in the outer layers of the neural tube (Fig. 3a, b), in the forebrain the first signs were detected at around E13.5 also in the outer surface of the neuroepithelium. The first signs of 1b transcript expression were detected in the lateral side of the lateral ventricle (Fig. 3d, e). During later embryonic development (E15.5), strong signal is detectable also in the lining of the aqueduct and in the deepest layers of the sensory region of the neocortex (S1, Fig. 3f). At E15.5, *Lsamp* 1a promoter is especially active in the caudate putamen (CPu, Fig. 3c), whereas this activity shades off during the first postnatal week (data not shown) and is not detectable in adult brain (CPu, Fig. 1f). The expression of *Lsamp* 1b promoter in the CPu is weak during development [as shown in E15,5; CPu (Fig. 3f) and moderate in adulthood (CPu, Fig. 1j)].

### Expression of *Lsamp* transcripts correlates with behavioral measures of trait anxiety and social interaction

**Fig. 4:**
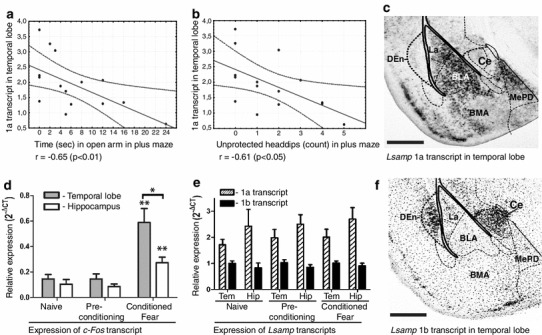
*Lsamp* expression in temporal lobe correlates with measures of trait anxiety (**a**, **b**), but was not altered 45 min after acute conditioned fear experience (**e**) that significantly raised *c*-*Fos* transcript in the temporal lobe and hippocampal area (**d**). Distribution of *Lsamp* 1a (**c**) and 1b (**f**) in the temporal lobe. *Dashed lines* indicate 95 % confidence limits (**a**, **b**). The *whiskers* represent SEM; *p < 0.01; **p < 0.05 (**d**, **e**). *BLA* basolateral amygdaloid nucleus, anterior part, *BMA* basomedial amygdaloid nucleus, anterior part, *Ce* central amygdaloid nucleus, *Den* dorsal endopiriform nucleus, *Hip* hippocampus, *La* lateral amygdaloid nucleus, *MePD* medial amygdaloid nucleus, posterodorsal part, *Tem* temporal lobe. *Scale bar* 500 μm (**c** and **f**)

We looked for the correlations between various behavioral parameters and the activity of *Lsamp* transcript expression in three brain areas (ventral striatum, hippocampus and temporal lobe). Most of the significant correlations emerged between *Lsamp* expression in the temporal cortex and behavioral parameters in the elevated plus maze (Table 1). Both *Lsamp* 1a and 1b transcript levels were negatively correlated with time on open arms in the elevated plus maze (Fig. 4a). Furthermore, *Lsamp* 1a transcript levels were negatively correlated with unprotected headdips in the elevated plus maze (Fig. 4b), with the number of open arm entries and with the ratio between open and closed arm entries. There was also a significant positive correlation between *Lsamp* 1a transcript levels and the latency to enter open arm. Additionally, *Lsamp* transcript levels in the hippocampus and ventral striatum correlated with behavioral parameters in the social interaction test. Both *Lsamp* 1a and 1b transcript levels were negatively correlated with the time that the mice spent sniffing the other animal. The time of anogenital sniffing positively correlated with *Lsamp* 1a activity in the ventral striatum.

**Table 1:** Correlation analysis between relative expression levels (2^−ΔCT^) of *Lsamp* 1a and 1b transcript in three brain areas and behavioral parameters in the motility box, elevated plus-maze and social interaction test

	Hippocampus		Temporal lobe		Ventral striatum	
	1A	1B	1A	1B	1A	1B
Motility box						
Move, s	−0.01	−0.06	0.11	0.06	0.12	−0.34
Distance, m	−0.06	−0.16	0.19	0.08	0.05	−0.30
Time center, s	−0.40	−0.36	0.43	0.36	0.05	0.03
Time corner, s	0.21	0.33	−0.38	−0.10	−0.13	0.17
Elevated plus maze						
Closed arm entries	−0.20	−0.43	0.17	−0.02	0.08	−0.14
Open arm entries	0.16	0.16	−0.57*	−0.40	0.01	−0.34
Ratio open/closed arm entries	0.08	0.13	−0.63 *	−0.38	−0.11	−0.34
Latency, s	0.03	−0.01	0.53*	0.22	−0.15	0.45
Time on open arms, s	0.24	0.15	−0.65**	−0.66**	0.14	−0.21
Protected headdips	0.17	0.08	−0.06	−0.06	−0.12	−0.26
Unprotected headdips	0.10	0.12	−0.61*	−0.50	0.20	−0.40
SAPs	−0.05	0.10	0.24	0.23	0.11	0.17
Social interaction test						
Anogenital sniffing, s	−0.10	−0.22	−0.09	−0.26	0.54*	0.11
Sniffing other body parts, s	−0.67**	−0.65*	0.06	0.15	−0.19	−0.07
Time of social sniffing, s	−0.51	−0.60*	−0.01	−0.09	0.14	−0.06

The behavioral measures have been presented in either counts, seconds (s) or meters (m)

* *p* < 0.01, ** *p* < 0.05 (Spearman’s rank-order correlation)

In the experiment for acute fear response, all the mice in the “Conditioned fear” group displayed an obvious fear reaction as evidenced by startling response and freezing (data not shown). The “Conditioned fear” group had significantly higher *c*-*Fos* expression in the amygdala [*F*_(2,13)_ = 11.6, *p* < 0.01] and hippocampus [*F*_(2,13)_ = 8.8, *p* < 0.01] than the “Naïve” or “Pre-conditioning” groups (Fig. 3d). Furthermore, in the “Conditioned fear” group, the *c*-*Fos* activation in the temporal lobe was significantly higher than in the hippocampus (*p* < 0.05), indicating specific activation of the amygdaloid area in response to conditioned fear. There were no statistically significant changes in the expression levels of *Lsamp* 1a of 1b transcripts at the 45 min timepoint (Fig. 3e).

## Discussion

Limbic system-associated membrane protein has been linked with a wide spectrum of psychiatric disorders in humans and with behavioral alterations in rodents. The limbic system-specific neuroanatomical distribution of the *Lsamp* transcript and protein has been described in various species (Chesselet et al. 1991; Côté et al. 1996; Reinoso et al. 1996; Yamamoto and Reiner 2005) and LSAMP immunostaining has been used as an anatomical marker of the limbic system (Prensa et al. 2003; Uroz et al. 2004). Here we provide first evidence that the activity profile of the two alternative promoters of the *Lsamp* gene has a heterogenous anatomical distribution in the developing and adult brain and the activity of these two promoters correlates with trait anxiety and social behavior in mice.

Limbic system-associated membrane protein 1a promoter is transcriptionally active in the “classic” limbic structures known to be especially important for emotional and motivational functions (Heimer and Van Hoesen 2006). Namely, 1a promoter is specifically active in the hippocampal formation, temporal cortex and amygdaloid area and also in the ventral striatum that includes nucleus accumbens and olfactory tuberculi; furthermore, 1a transcript is expressed specifically in the limbic, cingulate and insular cortex. Promoter 1a is active in the anterior thalamic nuclei that have been specified as “limbic thalamus” (Vogt and Gabriel 1993; Marchand et al. 2013) and in the anterior hypothalamus, including preoptic area that is a major interacting structure of the limbic system (Morgane et al. 2005). Promoter 1b of the *Lsamp* gene is notably active in the sensory pathways ranging from the brainstem and sensory nuclei in the thalamus and up to the primary sensory areas in the cortex. In the cerebral cortex, the signal from 1b promoter can be seen in layers 4 and 6 of the cortex (supplementary Fig. S1d) emphasizing systematic expression in the areas involved in the processing of sensory input. Layer 4 is the primary recipient of sensory input from the thalamus (Liao and Lee 2012) and most thalamic relay neurons receive feedback from layer 6 of the same cortical column they innervate (Lam and Sherman 2010). The specific activity of 1b promoter is obvious in the sensory pathways in visual, auditory and somatosensory areas. However, both 1a and 1b promoters are active in the neural pathways transmitting olfactory and gustatory information. This finding can be anticipated as brain regions associated with olfactory and gustatory perception (e.g., the piriform cortex and insular cortex) are often overlapping with brain regions that are involved in emotional processing (Gutman et al. 2013).

While *Lsamp* 1b promoter is predominantly active in sensory areas, it is also highly expressed in areas that are either traditional components of the limbic system and/or actively involved in regulating stress and arousal, such as the mammillary bodies and the paraventricular nucleus of the hypothalamus. Additionally, 1b promoter is prevalently active in the central nucleus of amygdala that is commonly referred to as the central part of the limbic structures (Heimer and Van Hoesen 2006). However, according to a recent study, the central nucleus of amygdala gets projections from several sensory-related regions (Bienkowski and Rinaman 2013). 1b promoter activity is highly enriched but not strictly limited to sensory areas; however, connections with the sensory systems can be found in most of the areas expressing 1b transcript (see supplementary Table S2 for overview). The expression of *Lsamp* in the brain areas processing sensory information has been reported in earlier studies (Reinoso et al. 1996; Yamamoto and Reiner 2005). Yet, the discussion of whether the distribution of LSAMP is really specific for limbic structures has not been raised. Our results indicate that it is questionable to use the summarized LSAMP staining as a marker of the limbic regions, however, we propose that *Lsamp* 1a transcript is intensively and specifically expressed in the brain areas that are commonly considered to be limbic structures (Heimer and Van Hoesen 2006; Morgane et al. 2005; Kötter and Stephan 1997).

Developmentally, the initial activation of both 1a and 1b promoters can be detected on embryonic day E12.5, the time preceding active neurite outgrowth and path-finding, suggesting that *Lsamp* is important already at the time of neuroepithelial patterning. The expressional initiation is consistent with previously published data about the developing brain of the rat (Pimenta et al. 1996). However, as in the adult, the expression pattern of the two alternative promoters differs remarkably at this early stage. Limbic system-associated membrane protein 1a promoter activation is initiated in the outer surface of the neuroepithelium. The first signs of 1b transcript expression, on the other hand, are detected in the lateral aspect of the lateral ventricle and in the lining of the aqueduct. According to our primary analysis, the extensive 1a transcript-specific staining in the dorsal striatum that disappears during the first postnatal week represents the most striking difference in the distribution of *Lsamp* between the developing and the adult brain. Strong 1a staining in the dorsal striatum before the second postnatal week overlaps with the time period of transient expression of LSAMP on developing axons (Horton and Levitt 1988) suggesting that *Lsamp* 1a promoter is specifically activated during the regulation of striatal development. The expression patterns of the *Lsamp* 1a and 1b transcripts during mouse embryogenesis indicate a potential role of the *Lsamp* gene and protein in the development all over the brain.

Increased levels of the *Lsamp* transcript have been associated with lower activity and higher levels of trait anxiety or acute fear reaction and the genetic deletion of the *Lsamp* gene in mice resulted in increased activity in novel environments and reduced anxiety (Catania et al. 2008; Innos et al. 2011). To get further insight of how LSAMP is involved in the regulation of adaptive and emotional behavior by the usage of alternative promoters, we studied behavioral correlates for *Lsamp* 1a and 1b transcripts in three brain areas. Most of the significant correlations appeared between *Lsamp* expression in the temporal lobe and behavioral parameters in the elevated plus maze. Higher levels of *Lsamp* 1a transcript had significant correlations with all of the measures indicating higher anxiety (Cruz et al. 1994) in the elevated plus-maze test. Higher levels of *Lsamp* 1b in the temporal cortex correlated significantly with the time that mice spent on open arm that is again, a common measure of anxiety. Current results are correlative in nature, but well in line with our previous loss-of-function studies with *Lsamp*-deficient mice displaying decreased anxiety (Innos et al. 2011).

In the current study, we did not detect any expressional changes in *Lsamp* transcripts after acute fear reaction although there is evidence that *Lsamp* is also activated in reaction to acute fear in the amygdaloid area of rats (Koks et al. 2004) and in the lateral amygdaloid nucleus of rats after fear conditioning (Lamprecht et al. 2009). It is most likely that acute changes are limited to specific subnuclei in the amygdala that can be more exactly separated from rat brain. In our study, we used the temporal lobe of mice, including all the amygdaloid nuclei and also the temporal cortex. However, the expression of *Lsamp* transcripts of the same area was significantly correlated with trait anxiety of the mice. We provide further evidence that the *Lsamp* gene is implicated in the formation of fear and anxiety processing circuits in the temporal cortex/amygdaloid area (Nieh et al. 2012), but this influence seems to be mediated differentially in acute fear reaction and trait anxiety. Although related, fear and trait anxiety are distinctly different—fear is an emotional reaction triggered by an immediate threat, while anxiety is a state of heightened apprehension in the absence of an immediate threat (Davis et al. 2010). Taken together, our results fit with previous evidence relating increased levels of *Lsamp* with heightened trait anxiety (Nelovkov et al. 2006; Alttoa et al. 2010); the implication of *Lsamp* in acute fear reaction seems to be more complicated and might be related with certain subnuclei in the amygdala or/and specific time points.

Both *Lsamp* 1a and 1b transcript levels in the hippocampus correlated negatively with social sniffing and *Lsamp* 1a transcript in the ventral striatum was positively correlated with the time of anogenital sniffing in the social interaction test. The implication of LSAMP in the regulation of social activity is again in line with the behavioral phenotype of *Lsamp*-deficient mice displaying lack of inter-male dominance hierarchy and whisker trimming (Innos et al. 2011). The positive correlation of *Lsamp* 1a transcript with anogenital sniffing fits with reduced anogenital sniffing accompanying reduced inter-male aggressiveness reported in *Lsamp*-deficient mice (Innos et al. 2011). General social sniffing is not altered in *Lsamp*-deficient mice; therefore, the correlation between higher level of *Lsamp* in the hippocampal area and shorter time of social sniffing may reflect higher *Lsamp* levels correlating with higher trait anxiety as the social interaction test was initially designed to measure anxiogenic and anxiolytic drug effects (File and Hyde 1978). Current data are in line with our previous reports showing that *Lsamp*-deficient mice have decreased anxiety and alterations in social behavior. Our results provide further evidence that *Lsamp* is functional in brain areas processing emotional reactions, particularly those related to anxiety/hyperactivity and social behavior.

Comparison of anatomical data from different species reveals high levels of conservation in the anatomical distribution of LSAMP transcript/protein. The summarized anatomical distribution from the current study is in line with the data from humans and primates: in humans LSAMP expression is intensive in the paraventricular thalamic nucleus (Uroz et al. 2004) and moderate in the nucleus accumbens and claustrum (Prensa et al. 2003). In primates, the hippocampus displays the strongest immunoreactivity, amygdala has a highly heterogeneous staining pattern (Côté et al. 1996) and ventral striatum displays more intense LSAMP immunostaining than the dorsal striatum (Côté et al. 1995). Furthermore, the twin promoter structure of the *LSAMP* gene seems to be essential also in humans. In the human genome, the exon 1a′ has been mutated by insertion of two nucleotides introducing a frame shift and resulting in a termination codon. Surprisingly, consequent loss of the acceptor site prevents the inclusion of the mutated exon 1a′ (Pimenta and Levitt 2004). The consequence of these two evolutionary events suggests that two promoters and alternatively regulated expression is needed for functional emotional responses in humans. Altered expression of *LSAMP* has been demonstrated in brain areas of human psychiatric patients in the frontal cortex (Behan et al. 2009) and hippocampus (Hyde et al. 1997), but in the previous studies 1a and 1b transcripts of the *LSAMP* gene in the nervous system have not been distinguished. Certain SNPs that reside in the first intron flanking exon 1b of *LSAMP* are associated with major depressive disorder (Koido et al. 2012). Furthermore, lower expression level of *LSAMP* 1a transcript has been linked with the susceptibility allele for coronary artery disease (Vance 2007). These data emphasize the importance of studying 1a and 1b isoforms separately to find out relevant information that can be used in diagnostic panels in the future.

The distinct system-specific use of alternative promoters reveals highly organized transcriptional regulation of LSAMP gene/protein associated with a broad spectrum of emotional behaviors. We propose that LSAMP is involved in emotional and social operating systems by complex regulation of two alternative promoters that guide the development of neural circuits in the limbic and sensory brain areas.

## Electronic supplementary material

Below is the link to the electronic supplementary material.

Supplementary material 1 (PDF 472 kb)

Supplementary material 2 (PDF 472 kb)
