# Supplementary material for: Lsamp is implicated in the regulation of emotional and social behavior by use of alternative promoters in the brain
Source: Brain Struct Funct. 2014 Mar 15;220(3):1381–93. doi: 10.1007/s00429-014-0732-x (PMC4409639; doi:10.1007/s00429-014-0732-x)
Supplement: Supplementary file 1 — Supplementary material 1 (PDF 472 kb) [file 429_2014_732_MOESM1_ESM.pdf]

Supplementary Fig. S1 (Philips et al. (maphilips@gmail.com) "*Lsamp* is implicated in the regulation of emotional and social behavior by use of alternative promoters in the brain")

Non-radioactive *in situ* RNA hybridization analysis representing the distribution of *Lsamp* 1a transcript (**a**,**e-g**,**k**,**q**,**t**) and universal *Lsamp* transcript (**b**) and X-Gal staining representing 1b promoter activity (**d**,**h-j**,**l-p**,**r**,**s**,**u**) and NeuN immunostaining (**c**).

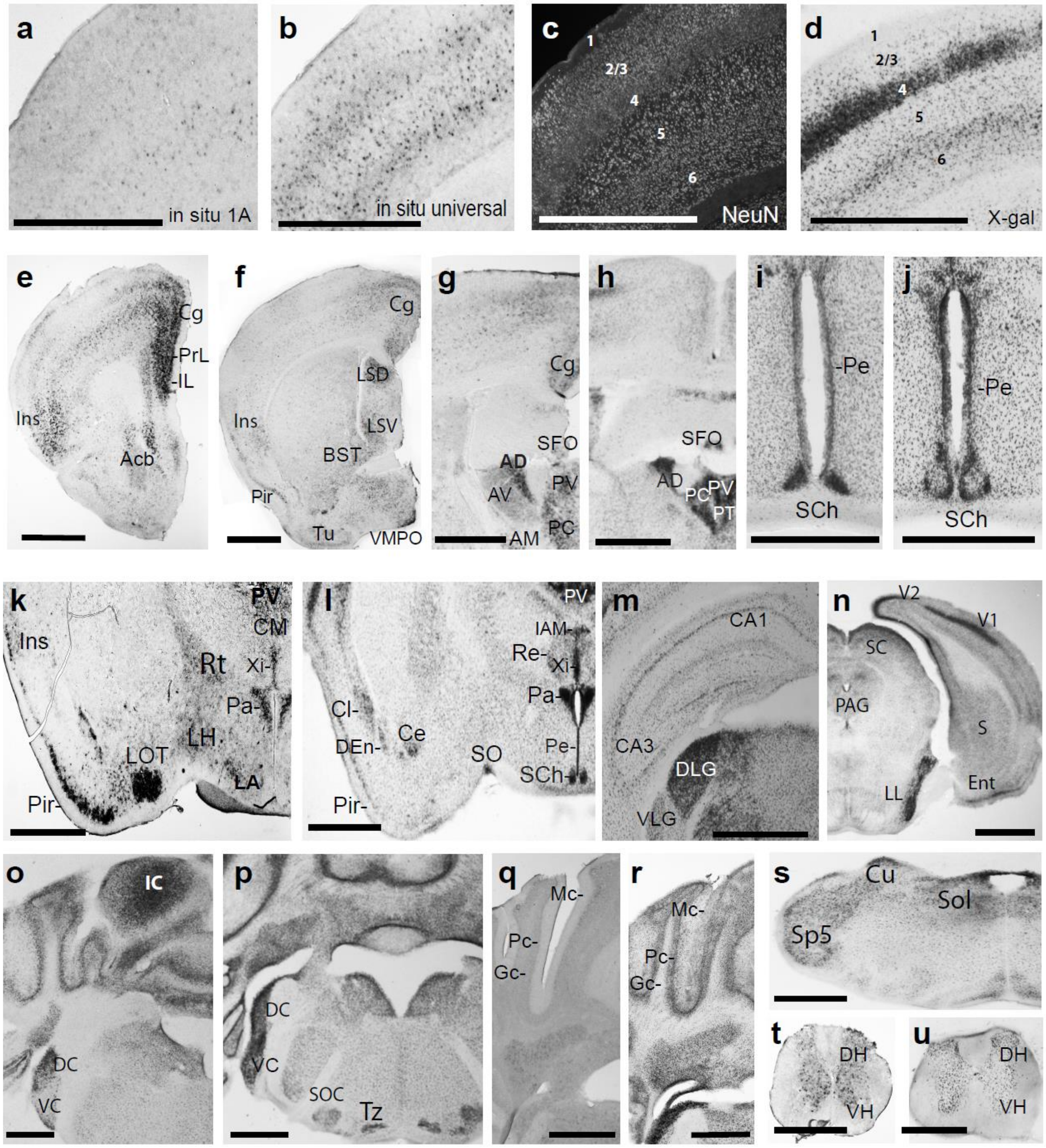

Supplementary Fig. S1 abbreviations: Acb (accumbens nucleus), AD/AM/AV (anterodorsal/ anteromedial/ anteroventral thalamic nucleus), BST (bed nucleus of stria terminalis), CA1/CA3 (CA1/CA3 field of hippocampus), Ce (central nucleus of amygdala), CM (central medial thalamic nucleus), CPu (caudate putamen), DEn (dorsal endopiriform nucleus), DLG/VLG (dorsal/ventral lateral geniculate nucleus), Cg (cingulate cortex), Cl (claustrum), Cu (cuneate nucleus), DC/VC (dorsal/ventral cochlear nucleus), DH/VH (dorsal/ventral horn of the spinal cord), Gc/Mc/Pc (granule/molecular/Purkinje cell layer of the cerebellum), IAM (interanteromedial thalamic nucleus) IC/SC (inferior/superior colliculus), IL (infralimbic cortex), Ins (insular cortex), LA/LH (lateroanterior/lateral hypothalamus), LSD/LSV (lateral septal nucleus, dorsal part/ventral part), LL (nuclei of lateral lemniscus), LOT (nucleus of the lateral olfactory dract), Pa/Pe (paraventricular/periventricular hypothalamic nucleus), PV (paraventricular thalamic nucleus), PAG (periaqueductal gray), Pir (piriform cortex), PrL (prelimbic cortex), PC (paracentral thalamic nucleus), PT (paratenial thalamic nucleus), Rt (reticular thalamic nucleus), Re (reuniens thalamic nucleus), SFO (subfornical organ), SCh (suprachiasmatic nucleus), Sol (solitary nucleus), Sp5 (spinal trigeminal nucleus), S (subiculum), SO (supraoptic nucleus), SOC (superior olivary complex), Tz (trapezoid body), Tu (olfactory tubercle), VMPO (ventromedial preoptic nucleus), Xiphoid thalamic nucleus (Xi). Scale bar = 1 mm.
