# Supplementary material for: Lsamp is implicated in the regulation of emotional and social behavior by use of alternative promoters in the brain
Source: Brain Struct Funct. 2014 Mar 15;220(3):1381–93. doi: 10.1007/s00429-014-0732-x (PMC4409639; doi:10.1007/s00429-014-0732-x)
Supplement: Supplementary file 2 — Supplementary material 2 (PDF 472 kb) [file 429_2014_732_MOESM2_ESM.pdf]

Supplementary Table S2 (Philips et al. (maphilips@gmail.com) "*Lsamp* is implicated in the regulation of emotional and social behavior by use of alternative promoters in the brain")

Distribution of *Lsamp* 1a, 1b and transcripts in the mouse brain. (1a+1b) summarized distribution refers to the staining with universal *Lsamp* probes that do not distinct different isoforms. The table represents distribution data from all the alternative staining methods that were used in the current paper. The intensity of the signal has been estimated on a scale: 0 – baseline; 1 – weak, 2 – modest, 3 – strong, 4 – very strong. The brain areas are categorized as sensory (auditory, visual, somatosensory, gustatory and olfactory) or "limbic". The brain areas were categorized as "limbic" according to Heimer and Hoesen (2006) and Morgane et al. (2005).

	**Area**	**Abbreviation**	**1a**	**1b**	**1a+1b**	
**FOREBRAIN**	Olfactory bulb	OB	1	1	1	Olfactory
	Cerebral cortex					
	Orbital cortex	LO, VO	2	0	1	Limbic
	Prelimbic/ Infralimbic cortex	PrL/IL	3	0	3	Limbic
	Dorsal peduncular cortex	DP	0	3	2	
	Cingulate cortex	Cg	4	0	2	Limbic
	Retrosplenial agranular cortex	RSA	0	2	2	
	Retrosplenial granular cortex	RSG	3	1	2	
	Granular insular cortex	G Ins	3	1	2	Gustatory/Limbic
	Agranular insular cortex	A Ins	3	0	2	Gustatory/Limbic
	Ectorhinal cortex	Ect	1	0	1	
	Entorhinal cortex	Ent	0	1	1	Limbic
	Claustrum	Cl	0	1	1	
	Dorsal endopiriform nucleus	DEn	0	2	2	
	Piriform cortex	Pir	3	2	3	Olfactory
	Temporal association cortex	TeA	2	1	2	
	Parietal association cortex	PtA	1	1	1	
	Primary auditory cortex	Au1	1	2	2	Auditory
	Secondary auditory cortex	Au2	1	2	2	Auditory
	Primary visual cortex	V1	0	3	2	Visual
	Secondary visual cortex	V2	0	1	1	Visual
	Primary Somatosensory cortex	S1	0	4	2	Somatosensory
	Secondary Somatosensory cortex	S2	1	3	2	Somatosensory
	Primary motor cortex	M1	1	1	1	Motor
	Secondary motor cortex	M2	1	0	1	Motor

Septal and basal forebrain regions

Medial septal nucleus	MS	1	0	1	
Lateral septal nucleus, dorsal part	LSD	2	3	3	
Lateral septal nucleus, ventral part	LSV	2	2	2	
Lateral septal nucleus, intermediate part	LSI	0	1	1	
Septofimbrial nucleus	SFi	2	3	2	
Subformical organ	SFO	1	2	2	
Bed nucleus of the anterior commissure	BAC	0	4	2	
Lateral bed nucleus of stria terminalis	BSTL	1	1	2	Limbic
Medial bed nucleus of stria terminalis	BSTM	3	1	3	Limbic

Basal ganglia and striatum

Olfactory tubercle	Tu	2	0	1	Olfactory
Nucleus of the lateral olfactory tract	LOT	3	0	2	Olfactory
Nucleus accumbens core	AcbC	3	1	1	Limbic
Nucleus accumbens shell	AcbSh	3	1	1	Limbic
Caudate Putamen	CPu	0	1	1	Motor
Globus pallidus	GP	0	1	1	Motor
Lateral stripe of striatum	LSS	0	3	1	

Hippocampal formation

CA1	CA1	4	1	4	Limbic
CA2	CA2	4	1	4	Limbic
CA3	CA3	4	1	4	Limbic
Dentate gyrus	DG	4	1	3	Limbic
Subiculum	S	2	0	2	Limbic
Amygdala					
Amygdalohippocampal area	AHi	4	0	3	Limbic
Intercalated nuclei of the amygdala	I	0	1	0	
Central amygdaloid nucleus	Ce	1	3	2	Limbic
Lateral amygdaloid nucleus	La	3	1	3	Limbic
Basolateral amygdaloid nucleus	BL	3	0	3	Limbic
Basomedial amygdaloid nucleus	BM	3	0	3	Limbic
Medial amygdaloid nucleus	MeA	3	1	3	Limbic
Medial amygdaloid nucleus, posterodorsal	MePD	2	1	3	Limbic
Medial amygdaloid nucleus, posteroventral	MePV	4	0	3	Limbic
Posterolateral cortical amygdaloid nucleus	PLCo	3	1	3	Limbic
Posteromedial cortical amygdaloid nucleus	PMCo	4	1	4	Limbic

DIENCEPHALON *Hypothalamus*

Medial preoptic nucleus	MPN	4	0	3	Limbic
Medial preoptic area	MPA	2	0	2	Limbic
Lateral preoptic area	LPO	2	0	2	Limbic
Periventricular hypothalamic nucleus	Pe	0	4	3	
Anteroventral periventricular nucleus	AVPe	0	4	3	
Lateral hypothalamus	LH	2	0	2	
Lateroanterior hypothalamus	LA	2	0	2	Limbic
Supraoptic nucleus	SO	0	2	2	
Suprachiamatic nucleus	SCh	0	4	2	
Paraventricular nucleus	Pa	3	4	4	Limbic
Medial Tuberal nucleus	Mtu	3	0	3	
Dorsomedial hypothalamus, compact	DMC	3	2	3	
Dorsomedial hypothalamus	DM	3	0	3	
Ventromedial hypothalamic nucleus, anterior part	VMH	0	2	2	
Ventromedial hypothalamic nucleus, ventrolateral part	VMHDM	3	0	3	
Ventromedial hypothalamic nucleus, dorsomedial part	VMHVL	3	0	3	
Arcuate nucleus	Arc	0	4	3	
Mammilary bodies	MB	4	4	4	Limbic
Ventral tuberomammillary nucleus	VTM	2	4	4	Limbic
Ventromedial preoptic nucleus	VMPO	3	0	3	

Thalamus

Anterodorsal thal. n.	AD	3	4	4	Limbic
Anteroventral thal. n.	AV	2	0	2	Limbic
Anteromedial thalamic nucleus	AM	1	0	1	Limbic
Paraventricular thal. n.	PV	2	3	3	
Ventrolateral thal. n.	VL	0	1	0	Motor
Ventromedial thalamic nucleus	VM	0	0	0	Motor
Ventral posterolateral nucleus	VPL	0	3	1	Somatosensory
Ventral posteromedial nucleus	VPM	0	3	2	Gustatory
Paratenial thal. n.	PT	1	4	3	
Mediodorsal thal. n.	MD	0	3	2	Olfactory
Laterodorsal medial nucleus	LD	0	2	1	Somatosensory
Central medial thal. n.	CM	2	0	1	
Paracentral nucleus	PC	1	2	1	
Parafascicular thalamic nucleus	PF	3	2	3	

Reuniens thalamic nucleus	Re	0	1	1	
Reticular thal. n.	Rt	2	0	2	
Xiphoid thalamic nucleus	Xi	1	2	2	
Interanteromedial thalamic nucleus	IAM	0	2	1	
Medial habenular n.	Mhb	4	4	4	
Lateral habenular n.	LHb	0	2	2	
Posterior thalamic nuclear group	Po	0	2	1	
Lateral posterior thalamic nucleus	LP	0	2	1	
Dorsal lat. Geniculate n.	DLG	0	4	1	Visual
Ventral lat. Geniculate n.	VLG	0	1	0	Visual
Medial geniculate n.	MG	0	3	2	Auditory
Subthalamic nucleus	STh	1	2	3	
Zona Incerta	ZI	0	0	0	

MIDBRAIN

Superior colliculus	SC	0	2	1	Visual
Inferior colliculus, central nucleus	IC	0	3	1	Auditory
Substantia nigra, pars reticulata	SNR	1	2	2	
Substantia nigra, pars compacta	SNC	1	2	2	
Periaqueductal gray	PAG	1	1	2	

BRAINSTEM

Nuclei of lateral lemniscus	LL	0	3	2	Auditory
Solitary nucleus	Sol	0	1	1	Gustatory
Superior olivary complex	SOC	0	1	1	Auditory
Nucleus of the trapezoid body	Tz	0	2	1	Auditory
Dorsal cochlear nucleus	DC	0	3	2	Auditory
Ventral cochlear nucleus	VC	0	3	2	Auditory
Cuneate nucleus	Cu	0	2	1	Somatosensory
Gracile nucleus	Gr	0	2	1	Somatosensory
Spinal trigeminal nucleus	Sp5	0	2	1	Somatosensory

CEREBELLUM

Molecular layer	Mc	0	1	1	
Granule cell layer	Gc	1	0	1	
Purkinje cell layer	Pc	0	3	2	

SPINAL CORD

Dorsal horn	DH	2	2	2	
Ventral horn	VH	2	2	2	
